# Micromechanical Study of Hyperacetylated Nucleosomes Using Single Molecule Transverse Magnetic Tweezers

**DOI:** 10.3390/ijms24076188

**Published:** 2023-03-24

**Authors:** Santosh Gaire, Roberto L. Fabian, Raghabendra Adhikari, Pamela L. Tuma, Ian L. Pegg, Abhijit Sarkar

**Affiliations:** 1 Department of Physics and Vitreous State Laboratory, The Catholic University of America, Washington, DC 20064, USA; 2 Department of Biology, The Catholic University of America, Washington, DC 20064, USA

**Keywords:** single molecule micromanipulation techniques, transverse magnetic tweezers, horizontal magnetic tweezers, proteins, DNA–protein complexes, DNA–histone interactions, nucleosomes, histone post-translational modifications

## Abstract

Nucleosomes are stable complexes of DNA and histone proteins that are essential for the proper functioning of the genome. These structures must be unwrapped and disassembled for processes such as gene expression, replication, and repair. Histone post-translational modifications (PTMs) are known to play a significant role in regulating the structural changes of nucleosomes. However, the underlying mechanisms by which these modifications function remain unclear. In this study, we report the results of single molecule micromanipulation experiments on DNA–protein complexes composed of hyperacetylated histone proteins using transverse magnetic tweezers. The experiments were conducted by pre-extending *λ*-DNA with a force less than 4 pN before introducing hyperacetylated histones into the sample chamber. The DNA shortened as the histones formed complexes with it and the nucleosome arrays were then exposed to increasing tension, resulting in quantized changes in the DNA’s extension with step sizes of (integral multiples of) ~50 nm. We also compared results of experiments using PTM histones and native histones with data collected for both types of histones for the same force ranges (2–80 pN) and loading rates. Our data show that hyperacetylated nucleosomes require an unbinding force of around ~2.5 pN, which is similar to that required for native histones. Moreover, we identified clear differences between the step-size distributions of native and hyperacetylated histones and found that in contrast to tethers reconstituted with native histones, the majority of nucleosomes in tethers compacted with hyperacetylated histones underwent disassembly at forces significantly lower than 6 pN.

## 1. Introduction

The diverse forms that protein post-translational modifications (PTM) take and the critical roles they play in the function of many proteins, including histones, make PTMs an important research topic. By covalently adding (or removing) a chemical group to an amino acid, it is possible to change the structure and functionality of many proteins. Multiple post-translational modifications of histones have been discovered as a result of a sustained research effort spanning multiple decades [1,2]. Among other PTMs, it is now known that histones can be acetylated, phosphorylated, ubiquitinated, and methylated at their N-terminal tails [3,4,5,6,7,8,9,10,11].

Histones play a key role in compacting DNA inside a cell nucleus, so it is not surprising to find that such histone tail modifications can affect higher-order chromatin architecture by changing DNA–histone interactions within and between nucleosomes [12,13,14,15,16]. Bulk assays have shown that acetylation of nucleosome core histones reduces the overall stability or binding affinity of the nucleosome to DNA [17,18,19,20]. Nevertheless, how, or how much, acetylation affects the stability of nucleosome is still not well understood.

In recent years, several studies have demonstrated that acetylation can directly alter the structure and dynamics of nucleosomes. Histone acetylation, which happens at a specific lysine on each of the four core histones, H2A, H2B, H3, and H4, is a modification that neutralizes the target lysine’s positive charge. Consequently, the DNA in nucleosomes constituted from post-translationally acetylated histones is bound more loosely to the core octameric particle, and the genetic machinery involved in, for instance, replication, transcription, translation, and DNA repair can access the bound DNA more easily [21,22,23,24,25,26].

The stability of nucleosomes can be modulated in multiple ways. However, histone modifications—in combination with destabilizing forces and torques applied by proteins bound to DNA segments near nucleosomes—may be key in altering the binding dynamics of DNA with the octameric core particle [27]. This could cause local chromatin unfolding, making previously inaccessible DNA available for genetic functions.

Single molecule techniques are a powerful method to explore the mechanical properties of individual protein–DNA complexes and especially the properties of short-lived intermediate states that are averaged in ensemble methods. Optical and magnetic tweezers and atomic force microscopy (AFM) are the mainstays of single molecule micromanipulation experiments and can also be combined with single-molecule fluorescence microscopy. For example, AFM enables the assessment of transient biomolecular conformations through accurate volumetric assays [28] and have been effectively employed to visualize assemblies formed between histones, DNA, and other proteins [29].

In this study, we investigate the response of reconstituted arrays of nucleosomes in which the histones were induced to have acetylation at every possible site (histone tails) of each of the core histones H2A, H2B, H3, and H4, a condition known as hyperacetylation [19,30]. Based on a review of the relevant literature, we have found no single molecule experiments micromechanically assaying the stability of nucleosome arrays reconstituted from hyperacetylated histones, especially in the low force regime. Magnetic tweezers operate naturally in the fixed-force ensemble (force is the control variable, extension is the measured or response variable,) and, compared to other single molecule techniques, have a much lower noise threshold. As a result, magnetic tweezers are capable of detecting unbinding events that would go unnoticed by other available techniques [31,32,33]. Here, we describe results from our studies of the mechanical response of single molecule DNA tethers compacted by hyperacetylated histones over a wide range of forces, with particular attention to low forces. Further, we describe data establishing that our findings are highly repeatable.

## 2. Results

### 2.1. A Typical Experiment Using Hyperacetylated Histones

In each experiment, we pre-extend the DNA by applying a force less than 4 pN prior to introducing hyperacetylated histones in the reaction chamber pre-loaded with λ-DNA and superparamagnetic beads. We introduce the histones at this force to thermodynamically bias the equilibrium towards the binding of the proteins to the tether; at higher forces, in particular, for forces larger than the critical force $f^{*}$ (the minimum amount of force required to initiate a decompaction of DNA-protein complex), binding is not favored. Following the introduction of the hyperacetylated histones, we can see the DNA shortening under tension as histones bind to the DNA and form DNA–protein complexes. We expose the nucleosome arrays to increasing tension after reconstitution. As the force rises, we can see quantized, step-like changes in the end-to-end extension of DNA, which, as we argue, correspond to the rupture of the individual (or integer multiples of) nucleosomes. For a detailed description of the experimental procedure and a schematic of the horizontal magnetic tweezers, see Section 4.

**Figure 1:**
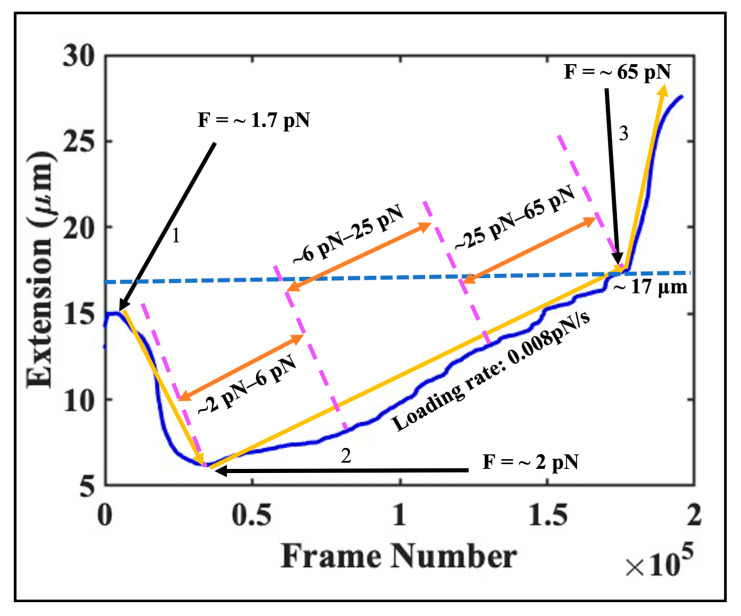
A plot of extension vs. time (number of frames) from a typical DNA–protein (hyperacetylated histones) interaction experiment. The tether extension of ~15 μm, corresponding to a force of ~1.7 pN, at which we injected hyperacetylated histones into the sample chamber using the buffer exchange approach is shown by arrow 1. The region between arrows 1 and 2 indicates the DNA’s compaction from ~15 μm to ~6.5 μm due to nucleosome array formation. The DNA extension increases from ~6.5 μm to ~17 μm as the force rises from arrows 2 to 3. The data above arrow 3 correspond to the DNA’s overstretching transition, which occurs around ~65 pN as expected.

Data from a typical experiment using hyperacetylated histones are plotted in Figure 1. In this experiment, we have introduced proteins into the sample cell by keeping the magnet position constant at a distance of ~1700 μm from the tethered, suspended bead; this position corresponds to a force of ~1.7 pN. A few minutes after protein injection, we visually observed compaction of the DNA molecule, from ~15 μm to ~6.5 μm. The compaction process was quite slow—it took ~4 min to observe full compaction. To ensure maximum nucleosome formation, we waited for an additional period of time—a few minutes—after no further tether compaction was visually apparent.

**Figure 2:**
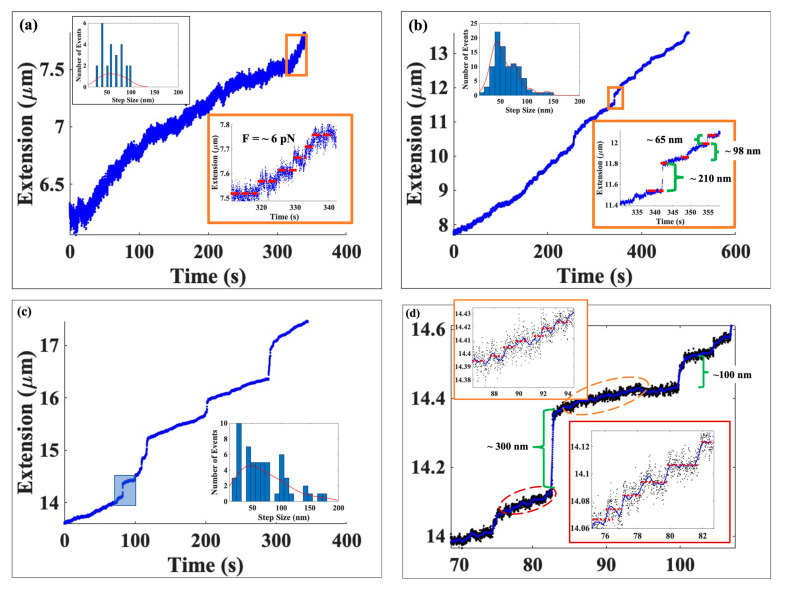
(**a**) Extension vs. time graph displaying data from about 6.2 μm to about 7.8 μm from Figure 1 corresponding to a force range we refer to as the low force regime. Starting with the DNA’s extension at ~6.2 μm, we began to increase the force by bringing the magnet nearer to the tethered, suspended bead. The force was gradually increased, causing the DNA to extend. The inset on the lower right of the figure shows the data for ~6 pN at a higher resolution. Although a staircase-type profile is clearly evident in the data, we extracted protein steps using a bilateral filter; the fits are shown by the red dashed lines in the inset. The histogram, which is shown in the upper left of the figure, depicts the total number of disassembly events detected in this force range. (**b**) plots extension as a function of time from ~7.8 μm to ~13.5 μm, corresponding to forces of ~6  pN to ~25 pN. These data are for the case where force is increased at a low loading rate, same as for the data in (**a**). Insets additionally show the zoomed-in area of the extension vs. time as well as the step-size histogram (right bottom and top left respectively). (**c**) presents data for DNA extensions between ~13.5 μm to ~17.4 μm from the experiment shown in Figure 1 when force was increased from ~25 pN to the point where the tether underwent the overstretching transition, ~65 pN. We observed 53 unbinding events, which are plotted in the step-size histogram—see inset at the bottom left. (**d**) shows a zoomed-in portion of the region within the shaded rectangle in panel (**c**). Further high-resolution data for portions of the data in panel (**d**) are given in the two insets. The lower right inset is taken from the lower dashed oval while the upper left inset present data from the upper dashed oval. In both sets, step-like features are clearly evident.

After the compaction process was completed, we began to increase the force by moving the magnet continuously toward the tethered bead at a very low speed of 1 μm/s (until the overstretching transition, where the loading rate was lowered to 0.5 μm/s.) The data between arrows 1 and 3 in Figure 1 encompass three distinct force regimes: low, from ~2 pN to ~6 pN; moderate, from ~6 pN to ~25 pN; and high, from ~25 pN and above. In Figure 2, we focus on data from Figure 1 spanning tether extensions from ~6.2 μm to ~17 μm.

In Figure 2a, we see that as tension was gradually increased from 2 to 6 pN, the DNA became extended in step-like, discrete increments from ~6.2 μm to ~7.8 μm. To better understand these events, we have magnified a portion of the figure at force ~6 pN, as shown in the inset. Although a staircase-type profile is visually evident, we wanted to quantify the data in an unbiased manner, and for that reason we used a bilateral filter to detect step-like features in the data. The red dashed lines in the inset to Figure 2a aid in distinguishing the disassembly events.

The saw-tooth-like fluctuations in the tether extension inside each plateau are likely due to extension changes resulting from local binding and unbinding of histone octamers and suggest that the tether re-extension is being achieved reversibly. One possibility is that while individual nucleosome core particles have been disassembled, the histone octamers may still remain (weakly) bound to small segments of the DNA. Subsequent thermal fluctuations in the tether can then re-establish, if only temporarily, one or more nucleosomes. However, this process has to be consistent with the overall value of the equilibrium extension at the given force.

We found that most steps were between ~35 nm and ~80 nm in length. In total, we observed 25 events in this force regime and most of them were centered around ~55 ± 7.8 nm. The total number of occurrences detected in this force regime has been displayed in the histogram—see the inset at the top left corner of Figure 2a.

Figure 2b is the graph of extension vs. time with the DNA extension changing from ~7.8 μm to ~13.5 μm. Here, the forces vary from ~6 pN to ~25 pN with the increase in force being achieved at the same, low loading rate as in the low force regime displayed in Figure 2a. The inset on the bottom right shows in higher resolution the extension trace with step like features clearly visible. Using the previously mentioned unbiased step-finding procedure based on a bilateral filter, we constructed a step-size histogram—upper left hand corner—for the entire data set displayed in Figure 2b (not just the boxed region). Although the bulk of the events centered around steps of length of ~55 nm, we observed a significant number of unbinding events with step lengths up to ~200 nm. We also note that in comparison to the low force regime, we observed a far greater number (96 events) of clearly delineated steps throughout this force range.

In Figure 2c we display data for the force range starting from 25 pN to where the magnetic bead became detached from the DNA molecule. The results shown in figure display a subset of the full data and span the range in DNA extension from ~13.5 μm to ~17.5 μm (the force ranges from ~25 pN to ~65 pN.) We were able to identify 53 unbinding occurrences in this portion of the data set. Of those, we found that 25% of the events had a step jump of less than 30 nm, with more than half of the occurrences having a mean step length of ~55 ± 5 nm. The remainder were centered around ~100.4 ± 6.1 nm and ~148 ± 5.9 nm resulting in a trimodal histogram—see histogram in the inset on the lower right side of Figure 2c The length of the DNA increases in multiples of ~50 nm, which is the expected signature of the mechanically induced removal of one (~50 nm extension increase), two (~100 nm extension increments), or more octamers bound to the λ-DNA tether.

Data from the shaded rectangle in Figure 2c are shown in more detail in Figure 2d. Of note, we found a large step with a length of ~300 nm, which was then followed by smaller jumps on both ends. The details of those small jumps are displayed in greater resolution in the insets in Figure 2d. The lower right inset shows the data depicted in the red dashed oval at the bottom while the upper left inset shows the data from the red dashed oval from the upper portion of the trace. In contrast to the low force regime, the plateaus in the insets in Figure 2d that lie between the steps do not show sawtooth-like structures. This suggests that nucleosomes have now been entirely disassembled and spontaneous thermal fluctuations of the tether are not sufficient to cause spontaneous reassembly by remaining bound octamer particles.

### 2.2. Comparison of the Mechanical Response of Native and Hyperacetylated Nucleosomes

In this section, we analyze and systematically compare the data for tethers compacted with native histones, previously reported in [34], with data for the nucleosomal arrays reconstituted from hyperacetylated histones.

#### 2.2.1. Comparison at Low Force Range

**Figure 3:**
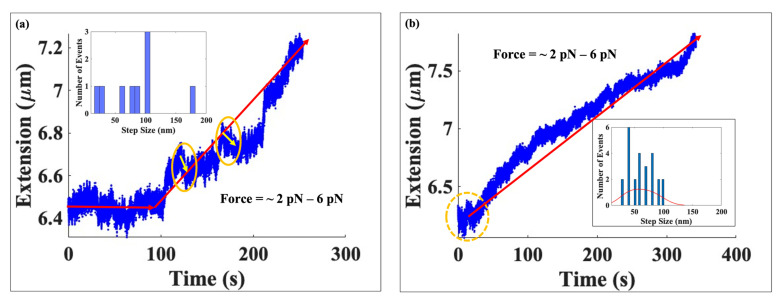
Two graphs of extension vs. time one for native (left) and another for hyperacetylated histones (right), in the low force range from ~2 pN to ~6 pN. (**a**) The extension of the DNA tether complexed with native histones showing the reconstituted chromatin extended from ~6.5 μm to ~ 7.3 μm. We found a total of nine unbinding occurrences, which are shown in the step-size histogram—see the inset in the left top corner. Arrows inside the solid yellow circles indicate DNA tether recompaction events in this force range, while the red lines are visual guides to the pattern of tether extension. (**b**) A single DNA tether bound with hyperacetylated histones subject to increasing forces from ~2 pN to ~6 pN as in (**a**) of this figure. The tether extension increased from ~6.3 μm to ~7.8 μm. In this force range, we found 25 discrete occurrences, which were used to construct the step-size histogram shown in the inset on the right bottom. The dashed circle shows the region where first unbinding event was observed, and the red line is a visual guide to the pattern of tether extension.

We begin with a brief review of the experimental approach. We manipulated both types of DNA–protein (native or hyperacetylated histones) complexes within a force window of ~2 pN to ~6 pN. In both studies, after introducing proteins into the sample cell, we began to increase the force after the DNA had achieved its maximum histone-mediated compaction. We used the same loading rate in both cases—magnet speed of 1 μm/s equivalent to ~0.008 pN/s except near the overstretching transition where it was decreased to 0.5 μm/s—to facilitate comparison between the two sets of findings. The data are shown in Figure 3.

Figure 3 compares the results from our experiments on single molecule DNA tethers compacted with either native or hyperacetylated histones and exposed to forces between ~2 pN and ~6 pN, which we refer to as the low force range. For DNA complexed with native histones, the tether extension increased from 6.5 μm to 7.3 μm; when bound to hyperacetylated histones, however, DNA extended from 6.4 μm to 7.8 μm, showing that at around 6 pN the tether compacted with hyperacetylated histones had a greater extension than the one complexed with native histones. This suggests that more of the hyperacetylated histones—and the nucleosomes they formed—were permanently ruptured when the 6 pN force was achieved compared to native histone-bound tethers. In the native histone case—Figure 3a—we only found nine disruption events while in Figure 3b of the same figure, corresponding to hyperacetylated histones, we obtained 25 events, almost three times more events compared to the native case. An interesting observation is that we noticed the onset of disassembly events in both cases to be around the same force of ~2.5 pN.

As the force was modulated upwards to 6 pN at loading rate of 0.008 pN/s, we observed that the DNA tether complexed with native histones did not change its extension for the first 120 s of the force being applied: the extension was constant at an average value of ~6.5 μm. In contrast, the extension of the DNA tether compacted with hyperacetylated histones increased continuously with time. For native histones, we only detected nine occurrences of disassembly: two were centered around ~22 nm, one was centered around ~50 nm, and the rest were centered around ~100 nm—see histogram in Figure 3a. Further, with native histones, we observed small reversals in extension, i.e., the tether spontaneously contracted by a small amount after each extension jump—see yellow circles in Figure 3a. This might be an indication of a greater energy barrier associated with the DNA-native histone complexes. In contrast, no extension reversals were observed for tethers bound to hyperacetylated histones. As plotted in the histogram in Figure 3b, for the hyperacetylated case we detected a total of 25 disassembly occurrences, of which more than 80% were centered around ~52 ± 7 nm.

#### 2.2.2. Comparison at Moderate and High Forces

**Figure 4:**
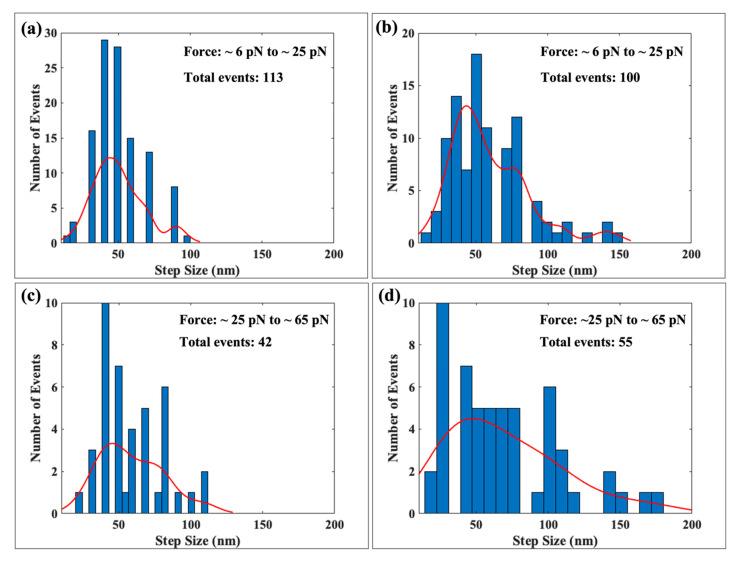
Step-size histograms comparing results obtained with native histones (**a**,**c**) to those obtained with hyperacetylated histones (**b**,**d**). (**a**,**b**) are for the moderate force range between 6 pN and 25 pN, while (**c**,**d**) for the high force range between 25 pN and 65 pN. The red curves are fits obtained using MATLAB.

We slowly ramped up the force on both types of tethers at a loading rate of 0.008 pN/s from ~6 pN to ~65 pN, the force where the DNA tether underwent the overstretching transition. To simplify the analysis, we have divided this force range into two parts which we label the moderate force range, from ~6 pN to ~25 pN, and the high force range from ~25 pN to ~65 pN. Here, we focus on our results for the step lengths for the two types of histones. In Figure 4, we show side-by-side the step-size distributions for DNA complexed with native versus hyperacetylated histones. Figure 4a,b of the figure present, respectively, the histograms comparing the two types of tethers—native and hyperacetylated—at the moderate force range, between ~6 pN and ~25 pN. Figure 4c,d report the step-size distributions for native and hyperacetylated histones respectively for the high force range, from ~25 pN to ~65 pN.

In the moderate force range (between ~6 pN and ~25 pN), we found 113 disassembly events for native histones, of which ~72 events, approximately 70%, were centered around ~52 ± 8.9 nm—see Figure 4a. We detected ~8 steps or ~7% of the total with a step length of ~90 nm. Most of the remaining steps were clustered around 20 nm with a few around 70 nm.

For the hyperacetylated case, we were able to detect a total of 100 rupture events within the same force range. We found three distinct peaks in the step length histogram centered around ~50 ± 7 nm (48 events, ~50% of the total), ~100 ± 5 nm (10 events, 10% of the total), and ~150 ± 5 nm (4 events, 4% of the total). The distribution of the remaining steps, about 36% of the total, did not display a prominent peak and was spread continuously with step lengths below ~30 nm. We note that in contrast to the native histone case, we detected steps over a wider range of step lengths and found a substantial number of occurrences with step lengths > 100 nm. The red curves in Figure 4a,b are histogram fits obtained using MATLAB and are presented as visual aids.

In the high force range, i.e., between ~25 pN and ~65 pN, we found 42 and 55 events for native and hyperacetylated cases respectively. For native complexes, most of the events were centered around ~50 ± 8.5 nm. Very few steps were seen centered around 100 nm. On the other hand, for hyperacetylated histone complexes, disassembly events were found to be distributed in a wide range of step lengths. Although a majority of the events were found to be clustered around ~50 nm, which is the length of DNA bound in a nucleosome, we also observed a significant number of events centered around ~30 nm, ~100 nm, and around ~150 nm. The 100 nm steps are consistent with the simultaneous rapture of two nucleosomes, whereas 150 nm steps could arise from the simultaneous rapture of three nucleosomes. The ~30 nm events are similar to what was found in optical tweezers studies of nucleosomes [35] and may involve the partial unwrapping of approximately one turn of the full one and three quarters turn of DNA wrapped around the histone octamer. The fits in red have been displayed to aid in the interpretation of the data.

## 3. Discussion

In this study we report results from single molecule micromanipulation experiments on DNA tethers complexed with native and post-translationally modified (PTM) histones. In a series of experiments, we allowed single DNA tethers kept under low tension to be compacted by either native or hyperacetylated histones. After the tether had compacted maximally, we increased the force and directly measured the minimum force $f^{*}$, at which the tether began to re-extend. Then, as we modulated the force upwards, we were able to distinguish individual re-extension steps, which we interpreted as arising from the force-mediated removal of one (or two or three or more) nucleosomes. We used a custom software [36] to identify and quantify in an unbiased manner these step-like features in the extension-vs-time plots.

The results reported here build upon previous research in our lab on custom horizontal magnetic tweezers. The design of the tweezers, as well as the steps we took to calibrate it, are described in detail in [37,38,39,40,41]. Extensive testing and calibration of the tweezers suggests that the single molecule data generated by the system are reproducible and free of systematic biases. Of direct relevance to the data reported here, we also previously carried out single-molecule experiments on DNA tethers complexed with native histones. The results of these experiments, which report of the mechanical stability of nucleosomes reconstituted from native histones on single DNA tethers, are discussed in [34], from which we draw the data used to make comparisons between results with native and hyperacetylated histones.

Our data allowed for a direct comparison of the mechanical stability of nucleosomes reconstituted from native or hyperacetylated histones. As hyperacetylation weakens the electrostatic attraction between the DNA and histones, we expected the DNA tethers compacted by hyperacetylated histones to display a clear mechanical signature of such post-translational modifications. We did see a difference in the mechanical response between the two types of tethers but, surprisingly, the same $f^{*}$ was obtained for both of them. The effect of hyperacetylating the histones was more subtle, at least in our micromechanical stability assay. The study also found that tethers compacted with PTM histones displayed a higher frequency of multi-step jumps compared to native histones in the same force range. The instability of the modified nucleosomes likely due to the charge neutralization on the lysines resulting from hyperacetylation. Co-operative interactions between neighboring PTM nucleosomes on the tether may also play a role in the rapid release of two or more nucleosomes simultaneously, suggesting that these interactions play a larger role in stabilizing arrays of hyperacetylated nucleosomes compared to native nucleosomes. The study also found differences in the stability of the nucleosomes, with PTM histones showing a rapid dissociation at or below 6 pN.

## 4. Materials and Methods

### 4.1. Micromanipulation Experiments with Horizontal (Transverse) Magnetic Tweezers

**Figure 5:**
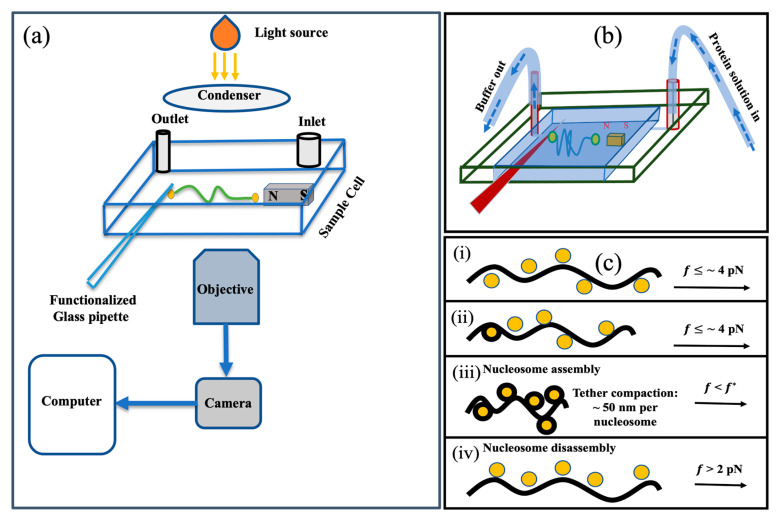
Schematic of our experiment. (**a**) The horizontal magnetic tweezers, modified from [41]. (**b**) The buffer exchange system. Sample cells have built-in outlet and inlet. The buffer is removed from the chamber through the outlet, while the protein solution is introduced via the inlet. The exchange rate is kept balanced by using two calibrated syringe pumps, one at each end. (**c**) The basic idea of DNA–histone interaction studies. (**i**) The first step is to introduce the protein solution into the sample cell (force ≤ 4 pN).The black curve represents the DNA tether, and the yellow solid circles represent histone octamers. (**ii**) Histone octamers begin to interact with DNA to form nucleosomes (force is still < 4 pN).(**iii**) Here, we pull the magnet further away from the tethered bead, dropping the force below 4 pN to allow more compaction events. (**iv**) This is the part of the experiment where we move the magnet toward the tethered bead leading to forces ranging from critical force $f^{*}$, i.e., ~2 pN, to several tens of pN upon the array.

We performed all single-molecule micromanipulation experiments using the horizontal magnetic tweezers (or transverse magnetic tweezers), discussed in references [39,41]—see schematics in Figure 5. In brief, we start by functionalizing λ-DNA (N3011S, New England Biolabs, Ipswich, MA, USA), which involves ligating biotinylated DNA oligomers (five biotins) complementary to 12 base polynucleotide overhangs to each end of the DNA molecule.

Next, we construct the sample cell which consists of a custom 3D-printed spacer, a bar magnet, and two coverslips. The two coverslips are adhered to the top and bottom of the spacer to create the sample cell’s ceiling and floor. The arrangement of the spacers also creates walls on three of the four sides of the sample cell; the remaining open side is used to insert and extract pipettes. In order to facilitate buffer exchange, the spacer features both an inlet and an outlet. The floor of the sample cell has a bar magnet attached to it using adhesive. After the sample cell is assembled, we incubate 3 μL of prewashed 2.8 μm diameter superparamagnetic beads (11205D, Thermo Fisher Scientific, Waltham, MA USA) coated with streptavidin with 1 μL end-functionalized λ-DNA in a working buffer. After incubation, 5 μL of the DNA–bead construct is injected into the sample cell, which also has a pre-inserted surface-functionalized glass pipette with its tip placed at a known reference location.

We then start looking for beads which are tethered to single DNA molecules. When a DNA-tethered bead is located, first we verify that the tether is indeed a single DNA molecule. To do so we increase the force on the tether by moving the magnet at a speed of 10 μm/s towards the suspended bead. The force is ramped up until the DNA overstretching transition is observed in the force range expected for a single DNA tether. Second, we expose the tether to a steady force of less than 4 pN for around 15 min in order to carry out a “No histone” control experiment. This eliminates the possibility of the buffer alone producing (or contributing in a non-trivial manner to) the observed compaction.

After confirming that a candidate tether is a single DNA molecule, we proceed to the second step, in which we introduce proteins—histones in our case—into the sample cell using a buffer exchange method. For details, see [34]. Briefly, we start by making the core histones and NAP1 solutions in the buffer of choice (in our case, 150 mM NaCl) and adjusting the final concentrations of core histones to ~0.05 mg/mL and NAP1 to ~0.1 mg/mL. We will refer to this as the “protein solution.”. Before adding the protein solution to the sample cell, we first let it incubate for one to two minutes at room temperature.

We now load the solution into sterilized Tygon tubing so that we may introduce the protein solution using the buffer exchange method. While introducing proteins into the sample cell, we normally maintain the magnet position ~1600 μm away from the tethered bead, which corresponds to a force of less than 4 pN. The custom designed buffer exchange system is used to extract buffer from the outlet while simultaneously pumping proteins in through the inlet in a balanced manner—see schematic in Figure 5b. Balanced exchange is achieved through the use of calibrated syringe pumps, one at each end. In order to avoid buffer overflow, the rates of the buffer solution entering and leaving the system are kept constant taking into account the differences in diameters of the inlet and outlet ports.

### 4.2. Histone Purification and Storage

Hepatic WIF-B cells culture and trichostatin A treatment was performed as previously described [42]. Histones were purified using histone purification mini kit (Catalog No. 40026, Active Motif, Carlsbad, CA, USA). Histone acetylation was determined by Western blot analysis using an antibody against acetylated histone 3 (Catalog No. sc-56616, Santa Cruz Biotechnology Inc., Dallas, TX, USA)

**Figure 6:**
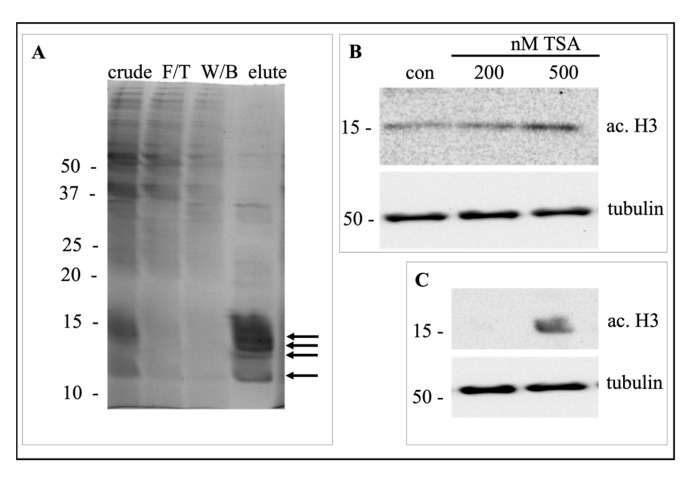
Native and acetylated histone purification. (**A**) Histones were extracted from a 100 mm tissue culture plate of WIF-B cells using histone purification mini kit. Crude histones, flow-through (F/T), wash buffer (W/B) and final elute (lane 1 to lane 4) were collected and loaded onto 15% SDS-PAGE. The gel was stained with Coomassie blue to visualize protein bands. The arrows on the right indicate bands resembling the molecular weight of H3, H2A, H2B, and H4 from top to bottom. (**B**) WIF-B cells were grown in 1 cm × 1 cm coverslips and treated with 200 or 500 nM trichostatin A (TSA) for 30 min. The whole cell lysate was made in 500 μL of Laemmli sample buffer. Ten μL of the lysate was immunoblotted for acetylated histone 3 (ac.H3) using Western blot. Tubulin was used as a loading control. (**C**) Ten μg of histones purified from control or 500 nM TSA treated WIF-B cells grown on 100 mm tissue culture plate were immunoblotted against ac.H3. The whole cell extract was immunoblotted against tubulin to use as a loading control. The numbers on left represent the molecular weight in KDa of the protein standard.

The histones were extracted and purified from WIF-B cells and validated by a Coomassie blue staining on SDS-PAGE. The presence of four bands between 10 and 15 KDa in both crude histone and final elute confirms proper histone purification (Figure 6A). As a measure of histone acetylation, histone 3 acetylation was determined. The darker immunoreactive bands for ac. H3 compared to control shows enhanced histone acetylation with 200 and 500 nM TSA (Figure 6B). Histone acetylation was further tested in purified histones. An immunoreactive band against ac.H3 was only detected in cells treated with TSA but not in control (Figure 6C), confirming our protocol of native and acetylated histone isolation.

Purified histones were than aliquoted into small tubes at 0 °C. The volume for purified protein aliquots was adjusted so that the addition of 100 μL of buffer yielded a concentration of 0.05 mg/mL of histones in solution. Similarly, NAP1 was aliquoted into small tubes at 0 °C and the volume of the NAP1 was adjusted so the addition of 100 μL of buffer resulted in a concentration of 0.1 mg/mL of NAP1 in solution. The aliquoted histones and NAP1 were stored at—80 °C.

### 4.3. Steps Detection and Data Analysis

Once data collection from a protein experiment was completed, we ran the recorded movie through a custom offline particle tracking algorithm. Next, using a custom step-finder algorithm [36], the nucleosomal rupture events were identified in the extension time-trace data. In brief, MATLAB code was developed to identify jumps in extension within the time traces. Each individual data set was filtered using a bilateral Gaussian kernel-type filter. Then, extensions were binned to form a distribution with bin height proportional to the number of successive frames so that an extension occurs in the filtered data set. The second derivative of this distribution was computed at each bin location, and the extension histogram was added to it. The result was then squared to avoid negative values; the local minima of the resulting distribution correspond to steps or jumps; the peaks corresponded to the plateaus or constant extension regions between successive jumps.

We created step distribution histograms in order to better understand the distribution of steps in our step extension profile data set. We fitted each histogram using the “histfit” function in MATLAB to help visualize the step heights and their distribution. The step distribution data, bin size, and distribution type “kernel” were among the parameters taken into consideration throughout the fitting procedure. We explicitly chose a bin size of 10 nm because the smaller bin sizes, such as ~1 nm for instance, did not improve the interpretability of the data since we were unable to resolve extension changes this small. On the other hand, we found that using larger bin sizes led to an artificial reduction in precision since many events that we could discriminate in the data were lumped together. The step-finder algorithm rounds the measured steps to a nearest multiple of 5 nm. The bin-size for counting these step events was 10 nm.

## 5. Conclusions and Future Perspectives

This study reports on the characteristics of nucleosomes formed using native or hyper-acetylated histones. We use custom horizontal magnetic tweezers to apply tension to nucleosomes while they form and to mechanically disassemble them. The study finds that the measured critical force (the minimum force at which a nucleosome is mechanically disassembled) is in agreement with theoretical predictions $f^{*}$, for both native and hyperacetylated histones. However, the pattern of disassembly is markedly different for the two types of nucleosomes as quantified by the step-size distributions for nucleosomes reconstituted from native and PTM histones. Future single molecules experiments can map out the contributions to the binding stability of nucleosomes of post-translational modifications to specific residues of the constituent histone proteins.

Our study highlights the kinds of data that can only be obtained from single molecule approaches. Moreover, experimental designs such as ours can be used to study other DNA–protein interactions and especially the binding to DNA to histone octamers with other types of post-translational modifications. For instance, one can explore the effect of hypo-acetylation on chromatin formation, as well as modifications to specific lysines in specific histones. Novel DNA tethers can also be used to modulate the electrostatic attraction between octamers and DNA. Compared to optical tweezers, magnetic tweezers are relatively easy to implement and operate, while yielding data sets with force and spatial resolutions comparable to those from optical tweezers (although optical tweezers have higher temporal resolution.) Building on studies such as ours, new experiments could mechanically assay the binding of multimeric protein complexes to DNA in a dynamic manner, with the individual complexes being built up one component protein at a time on the tether itself. These and other extensions to our experiments open up exciting opportunities to study biomolecular machines in unique ways not possible using ensemble approaches, while using a relatively simple experimental apparatus.

## Data Availability

The data presented in this study are available on request from the corresponding author.
